# Health related quality of life of tuberculosis patients in South India: A longitudinal assessment study

**DOI:** 10.1371/journal.pone.0328484

**Published:** 2025-07-23

**Authors:** Vasantha Mahalingam, Muniyandi Malaisamy, Bella Devaleenal Daniel, Adhin Bhaskar, Lavanya Jayabal, Lakshmi Murali, Bharadhidasan Palani, Kavi Mathiyazhagan, Sai Madhu Yadla, Padmanaban Srinivasan, Ponnuraja Chinnaiyan

**Affiliations:** 1 Department of Statistics, ICMR-National Institute for Research in Tuberculosis, Chennai, Tamil Nadu, India; 2 Department of Health Economics, ICMR-National Institute for Research in Tuberculosis, Chennai, Tamil Nadu, India; 3 Department of Clinical Research, ICMR-National Institute for Research in Tuberculosis, Chennai, Tamil Nadu, India; 4 District Tuberculosis Centre, National Tuberculosis Elimination Programme, Chennai, Tamil Nadu, India; 5 State TB Training and Demonstration Centre (STDC), Chennai, Tamil Nadu, India; The University of British Columbia, CANADA

## Abstract

**Background:**

In India, there is no information on health related quality of life (HRQoL) of patients with drug sensitive tuberculosis (TB) using a longitudinal design that includes post- treatment period. This study is the first of its kind in India to assess HRQoL of TB patients from a longitudinal prospective and to identify the factors associated with changes in HRQoL.

**Methods:**

The study participants were 180 newly diagnosed drug-sensitive smear-positive pulmonary TB patients who were initiated on treatment under the National TB Elimination Programme (NTEP) in Chennai and Tiruvallur districts of Tamil Nadu, South India. The patients were interviewed at four different time points between 2020 and 2023 using validated questionnaires assessing general health (European Quality of Life-5 Dimensions-5 Level (EQ-5D-5L), Short Form health survey (SF-20)), disease specific (St. George’s Respiratory Questionnaire (SGRQ)) and mental health including depression and anxiety (Patient Health Questionnaire (PHQ-9), Generalized Anxiety Disorder (GAD-7)). The Friedman test was used to identify changes in HRQoL scores over time and generalised estimating equation (GEE) were applied to identify factors associated with HRQoL.

**Results:**

HRQoL scores of TB patients, as measured by different scales showed significant improvement from treatment initiation to treatment completion. The GEE analysis showed that the EQ-5D-5L scores over follow-up visits were significantly lower in females (−0.038, p < 0.005) and higher in those who did not skip their main meal in a day (0.077, p < 0.001). The PHQ-9 and GAD-7 scores were significantly higher among females (0.609, p < 0.05; 0.531, p < 0.05). Additionally, PHQ-9 scores were also higher among patients from rural district (0.392, p < 0.05). The SF-20 scores were significantly lower in patients aged >45 years (−1.675, p < 0.05), female (−3.809, p < 0.001) and unemployed (−2.277, p < 0.005). The SGRQ scores were higher in patients aged >45 years (3.043, p < 0.01), females (4.256, p < 0.05) and those from rural district (2.219, p < 0.05). The HRQoL scores were significantly higher in patients who did not skip their main meal and lower in females irrespective of the scales used.

**Conclusion:**

The HRQoL of TB patients improved significantly over a period of treatment. Gender, age, skipping main meals, region and employment status were the key factors influencing HRQoL. Focusing on HRQoL assessment in the care of TB patients could help to minimize physical, mental and social challenges and enable them to lead a normal life.

## 1. Introduction

Tuberculosis (TB) is one of the lung related airborne infectious diseases caused by *Mycobacterium* TB. It continues to be a major public health problem globally, especially in high burden countries like India, where socio-economic factors exacerbate the disease’s impact on patients’ physical and mental well-being. Globally, 8.2 million people were reported as newly diagnosed with TB in 2023, an increase from 7.5 million in 2022 [1]. India accounted for about 2.23 million of these cases in 2023, down from 2.42 million in 2022 [2]. The National TB Elimination Programme (NTEP), formerly known as the Revised National TB Control Program (RNTCP), is a key public health initiative by the Government of India that coordinates anti-TB activities across the country. The primary goal of NTEP is to reduce TB incidence and mortality, achieving early diagnosis, universal access to high-quality care, and ensuring complete treatment to prevent relapse. To achieve this goal, the NTEP has made significant efforts to control TB and provide free treatment to all TB patients. Furthermore, the National Strategic Plan outlines solutions and allocates resources to significantly reduce TB prevalence in the country [3].

Despite this progress, eliminating TB remains challenging due to social and economic barriers that affect treatment adherence, such as poverty and stigma, which contribute to non-adherence to treatment and recurrence of TB. The stigma associated with TB, combined with the long duration of treatment, often leads to social isolation, psychological distress, and economic hardships for patients. These factors can undermine patients’ overall well-being, affecting their ability to reintegrate into society and resume normal life. While the effectiveness of TB treatment is primarily evaluated based on clinical measures such as bacteriological confirmation, it is equally important to consider the other dimension of TB treatment outcomes, particularly Health Related Quality of Life (HRQoL). Understanding and improving HRQoL is essential, as it offers valuable insights into the well-being and overall satisfaction of TB patients and their families.

Previously published research has paid little attention to studying the HRQoL of TB patients in different dimensions such as physical, mental and social well-being [4]. Due to the nature and symptoms of the disease such as continuous cough, loss of appetite, weight loss, chest pain and breathing problems, TB patients feel tired and fatigued most of the time [5]. The disease also causes body pain, stigma, depression and emotional problems. In addition, TB can affect patients’ life even after successful treatment and it has an adverse effect on their long-term survival rates [6]. A longitudinal multi-centric study from South Africa evaluated changes in HRQoL among TB patients during the standard TB treatment course of six months using four different instruments [7]. Another study conducted in Nigeria had studied the variation in HRQoL among pulmonary TB patients during the intensive phase (IP) of treatment [8]. A study conducted among TB patients of Thai-Myanmar migrants reported that there was continuous improvement in HRQoL during treatment despite being low at the time of diagnosis. They applied a generalised estimating equation (GEE) method to assess the longitudinal changes in HRQoL of the patients [9]. Since GEE offers a powerful framework to investigate the evolving nature of the longitudinal data, it is widely applied to study the HRQoL over time of various diseases. Though there are many cross sectional studies that have measured the HRQoL of TB patients [4], only a few studies have used a longitudinal design to assess HRQoL of TB patients at different time points until treatment completion [10]. From our literature search, there is no information on changes in HRQoL of TB patients from the initiation of treatment to the post treatment period in India. In the current study, we assessed the changes in HRQoL at four different time points among new TB patients treated under NTEP using different tools. The objective of using multiple instruments for measuring HRQoL in our study was to capture a comprehensive and multidimensional view of the patient’s well-being. By incorporating both general and disease-specific tools, we aimed to assess various domains of health, including physical, psychological, and social aspects. The use of the European Quality of Life-5 Dimensions-5 Level (EQ-5D-5L) and Short Form health survey questionnaire (SF-20) enabled a broad evaluation of general health status, while the St George’s Respiratory Questionnaire (SGRQ) provided a focused assessment of respiratory-specific symptoms and their impact on HRQoL. Additionally, the inclusion of the Patient Health Questionnaire (PHQ-9) and Generalized Anxiety Disorder (GAD-7) enabled us to measure mental health aspects, specifically depression and anxiety, which are known to significantly affect HRQoL. This multi-instrument approach ensured a thorough evaluation, offering insights into how different aspects of health interact and contribute to overall HRQoL during and post-treatment. Specifically, the longitudinal evaluation of HRQoL enabled us to track changes over time, providing insights into how the patients’ well-being fluctuates during and after treatment. This study is the first of its kind in India to assess HRQoL of TB patients from a longitudinal prospective and to identify the factors associated with changes in HRQoL.

## 2. Methods

### 2.1 Study settings

Under the NTEP, new drug-sensitive TB patients are treated with a six month regimen consisting of 2 months of intensive phase (IP) with isoniazid (H), rifampicin (R), pyrazinamide (Z) and ethambutol (E), followed by 4 months of H, R and E (2HRZE/4HRE) as per five weight band categories [11]. The study population included TB patients who were registered for treatment under NTEP in Chennai and Tiruvallur districts of Tamil Nadu, South India. The recruitment of study participants was initiated in November 2020 and enrollment was completed in April, 2022.

### 2.2 Study design

This study was a prospective longitudinal study conducted among new TB patients.

### 2.3 Study participants

The newly diagnosed pulmonary drug-sensitive smear-positive TB patients who were registered for TB treatment under NTEP were screened for eligibility to enrol in the study.

#### Inclusion Criteria.

• Adult new pulmonary TB patients aged ≥18 years
• Patients willing to provide written informed consent

#### Exclusion Criteria.

• Sick and moribund patients
• Unstable domiciliary status (individuals or family lacking a permanent or stable place of residence or frequent relocations or homelessness, or living in temporary or inadequate housing conditions)
• A history of psychiatric illness
• HIV positive individuals
• Extra pulmonary TB patients
• Drug-resistant TB patients

### 2.4 Sample size

Assuming a 4-point change in the physical component mean score between baseline and sixth month of treatment with a corresponding standard deviation of 7, a one sided 5% level of significance and 95% power, the minimum sample size required was 36. The sample size was projected to be 80, accounting for a design effect of 2 and 90% coverage. A total of 160 patients were required, with 80 patients from a rural district and 80 patients from an urban district.

### 2.5 Tools for data collection

In the current study, we used five different components of HRQoL measurement scales, including two general health assessment tools: EQ-5D-5L version 3.0 including the EQ Visual Analogue Scale (EQ-VAS) and the 20-item Short Form Survey (SF-20); disease status assessment tool (SGRQ); depression and anxiety symptoms assessment tools, namely PHQ-9 and GAD-7. These tools are recommended for measuring HRQoL across different domains (S1 Appendix) [7,12]. These scales are widely used in Indian settings, have been validated in Tamil language and have been used in other studies [13–19]. In addition, patients’ demographic, socio-economic, life style characteristic, as well as body mass index (BMI) and clinical profiles were collected using a pre-coded semi-structured interview schedule.

### 2.6 Ethical consideration

The Institutional Ethics Committee (ICMR-NIRT IEC No. 201828 dated 23.01.2019) of the ICMR-National Institute for Research in Tuberculosis (NIRT) approved the study. All eligible TB patients were informed about the study objectives, duration and procedures and purpose in the local language. Additionally, a patient information sheet was given to ensure that participants clearly understood the study. After addressing their queries, a trained field investigator obtained written informed consent from the TB patients for participation. During the interview, the investigators provided counselling to ensure treatment compliance. TB patients with moderate to high depression and anxiety scores were appropriately referred for further care and management.

### 2.7 Data collection

Line listings of TB patients were prepared from TB registers maintained at the health facilities. Participants were recruited consecutively from multiple TB Units (TUs) in rural, Tiruvallur district and in urban, Chennai district. Trained study team members interviewed the enrolled TB patients and collected information at the treatment centres. The patients were interviewed at four different time points (i) Base line (within one week of initiation of treatment), (ii) at the end of IP of treatment, (iii) at the end of continuation phase (CP) of treatment, and (iv) after three months of completion of treatment. Post treatment follow-up of the patients for this study was completed in January, 2023. The data were collected in a portable data collection assistant (PDA) and were simultaneously synchronized in REDCap software on the same day of interview. The study team at ICMR-NIRT verified data accuracy and completeness. If any incompleteness was found in the data, it was rectified within 2–3 days. The study investigators supervised the data collection team and conducted random checks to ensure data quality. The recruitment of participants was stopped upon reaching 90 participants in each district. The data were cleaned and analysed using IBM SPSS Statistics software version 25.0.

### 2.8 Data analysis

The EQ-5D-5L index scores were calculated based on Indian set of weights [20]. The index scores of SF-20 [21] and SGRQ [22] were estimated using scoring guidelines provided by the respective tool developers. The standard scoring methods for the PHQ-9 and GAD-7 were used to calculate total scores for each patient [23,24]. The Friedman test which is a non-parametric statistical test was applied to assess changes in HRQoL of the TB patients over time using different tools for assessing general health, depression, anxiety and disease specific outcomes. The study participants who were lost-to-follow-up for the interview were considered to have data missing completely at random. The patients who attended only the baseline interview were excluded for further analyses. GEE is used to estimate population-averaged effects in longitudinal data, especially when responses within individuals are correlated across time points. However, GEE assumes that any remaining missing data at subsequent time points is either missing completely at random (MCAR) or missing at random (MAR) [25]. Time was included as a variable in the model to account for changes in HRQoL throughout TB treatment. This allowed us to examine the trajectory of HRQoL whether it improved, deteriorated, or remained stable over time. Hence, in this study, we applied the GEE model to assess the changes in HRQoL and to identify factors associated with HRQoL over different time points.

## 3. Results

### 3.1 Sample coverage

**Fig 1:**
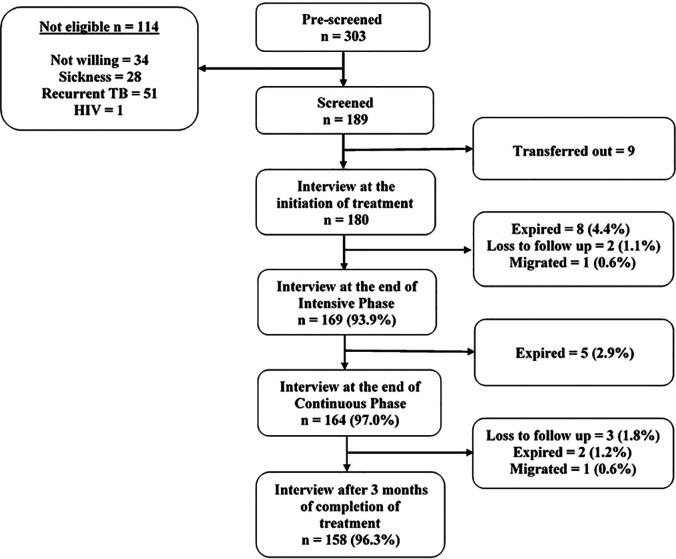
Recruitment of enrolment and follow-up status of study participants.

A total of 303 TB patients who started treatment at NTEP centers of Chennai (urban) and Tiruvallur (rural) districts were listed for screening. Among them, 114 were not eligible due to various reasons such as unwillingness to participate in the study, sickness, prior TB treatment, recurrent TB and HIV. The remaining 189 TB patients were screened for eligibility, 180 were enrolled in the study (Chennai = 90 and Tiruvallur = 90) and 9 (Chennai = 4; Tiruvallur = 5) were enrolled but later transferred to other districts (Fig 1). In the Chennai district, 90 (100%), 84 (93.3%), 81 (90%), and 75 (83.3%) patients were interviewed at the initiation of treatment, at the end of IP, at the end of CP and after three months of treatment completion respectively. Fifteen (16.7%) patients were not interviewed during follow-up due to 9 deaths (10%), 5 lost to follow-up (5.6%) and one migration (1.1%). In Tiruvallur district, 90 (100%), 85 (94.4%), 83 (92.2%) and 83 (92.2%) patients were interviewed at the same four time points. Seven (7.8%) patients were not interviewed during follow-up, including 6 deaths (6.7%) and one migrated (1.1%).

### 3.2 Demographic and socio-economic profile of the patients

Of 180 TB patients, 126 (70%) were males; 54 (30%) were females and 92 (51.1%) were > 45 years of age. Regarding socio-economic characteristics, 148 (82.2%) were literates, 32 (17.8%) illiterates; 122 (67.8%) employed and 58 (32.2%) unemployed (Table 1). Educationally, 39 (21.7%) had completed primary schooling, 47 (26.1%) completed middle school, 40 (22.2%) studied up to high school, 8 (4.4%) completed higher secondary education, 9 (5%) had a graduate degree, 2 (1.1%) had a post-graduate degree and 3 (1.7%) completed other diploma courses. The average monthly income of the patients and families was ₹7,110 and ₹12,147 respectively. The average monthly food expenditure of the family was ₹4,727. Ninety seven (53.9%) patients had a monthly income of ≤₹7,000, 102 (56.7%) had a family monthly income of ≤₹12,000 and 92 (51.1%) had a family monthly food expenditure of ≤₹4,500. Sixty- four (35.6%) patients reported consuming two main meals in a day, 103 (57.2%) had three main meals in a day and 117 (65.0%) patients had skipped meals sometimes. A total of 157 (87.2%) had a BMI < 23 kg/m^2^ indicating below normal weight. Regarding life style characteristics, 70 (38.9%) were smokers and 99 (55%) reported alcohol consumption.

**Table 1:** Basic characteristics of newly diagnosed pulmonary drug-sensitive smear-positive tuberculosis (TB) patients.

Variables	Chennai n (%)	Tiruvallur n (%)	Total n (%)
**Gender**			
Male	67 (74.4)	59 (65.6)	126 (70.0)
Female	23 (25.6)	31 (34.4)	54 (30.0)
**Age in years**			
≤45 years	39 (43.3)	49 (54.4)	88 (48.9)
>45 years	51 (56.7)	41 (45.6)	92 (51.1)
**Education**			
Literate	78 (86.7)	70 (77.8)	148 (82.2)
Illiterate	12 (13.3)	20 (22.2)	32 (17.8)
**Occupation**			
Employed	66 (73.3)	56 (62.2)	122 (67.8)
Unemployed	24 (26.7)	34 (37.8)	58 (32.2)
**Individuals’ monthly income**			
≤ ₹ 7,000	46 (51.1)	51 (56.7)	97 (53.9)
> ₹ 7,000	44 (48.9)	39 (43.3)	83 (46.1)
**Monthly income of family**			
≤ ₹ 12,000	49 (54.4)	53 (58.9)	102 (56.7)
> ₹ 12,000	41 (45.6)	37 (41.1)	78 (43.3)
**Monthly food expenditure for family**			
≤ ₹ 4,500	44 (48.9)	48 (53.3)	92 (51.1)
> ₹ 4,500	46 (51.1)	42 (46.7)	88 (48.9
**Ever smoked**			
Yes	34 (37.8)	36 (40.0)	70 (38.9)
No	56 (62.2)	54 (60.0)	110 (61.1)
**Ever consumed alcohol**			
Yes	49 (54.4)	50 (55.6)	99 (55.0)
No	41 (45.6)	40 (44.4)	81 (45.0)
**No of main meals in a day**			
1	6 (06.7)	5 (05.6)	11 (06.1)
2	32 (35.5)	32 (35.6)	64 (35.6)
3	51 (56.7)	52 (57.8)	103 (57.2)
≥4	1 (01.1)	1 (01.1)	2 (01.1)
**Skipping meals**			
Never	24 (26.7)	26 (28.9)	50 (27.8)
Sometimes	60 (66.7)	57 (63.3)	117 (65.0)
Often	6 (06.7)	7 (07.8)	13 (07.2)
**Body Mass Index (kg/m^2^)**			
<23	75 (83.3)	82 (91.1)	157 (87.2)
≥23	15 (16.7)	8 (08.9)	23 (12.8)
**Total**	90 (100)	90 (100)	180 (100)

### 3.3 Overall HRQoL

**Fig 2:**
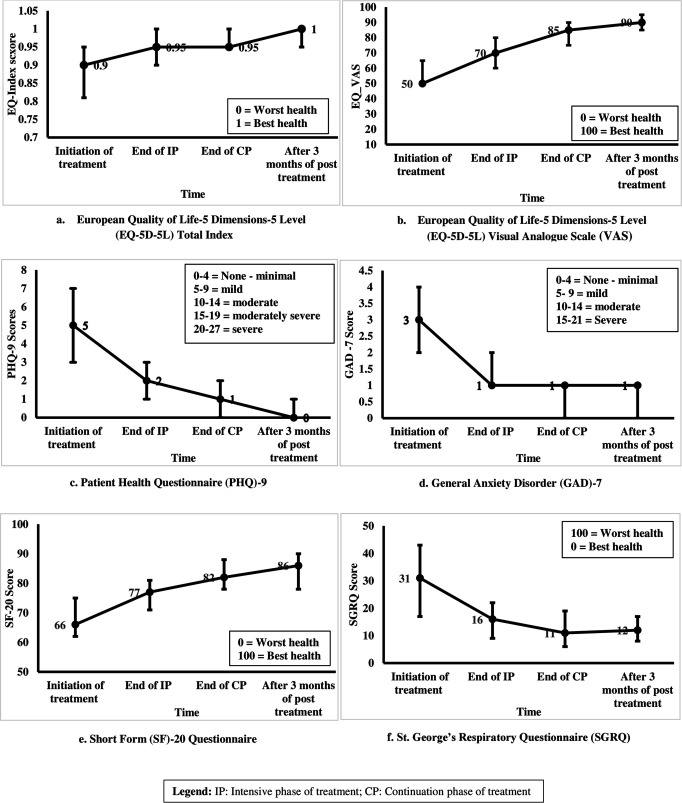
Changes in HRQoL of TB patients during and after treatment period.

The trend in overall HRQoL scores of the TB patients at different time points during and after treatment period is shown in Fig 2. The changes in scores were found to be statistically significant during and after treatment using Friedman test (Table 2).

**Table 2:** Comparison of health related quality of life (HRQoL) scores among tuberculosis (TB) patients over time using different Assessment tools (n = 158).

HRQoL Domains	Mean (Standard Deviation)	Median (Inter Quartile Range)	p-value
**European Quality of Life-5 Dimensions −5 Level** **(EQ-5D-5L) Visual Analogue Scale (VAS) score**			
At the initiation of treatment	55.76 (11.95)	50.00 (50.00 - 65.00)	<0.001
At the end of Intensive Phase (IP)	71.09 (11.13)	70.00 (60.00 - 80.00)	
At the end of Continuation Phase (CP)	82.76 (10.46)	85.00 (75.00 - 90.00)	
After three months of completion of treatment	88.25 (9.26)	90.00 (85.00 - 95.00)	
**EQ-5D-5L Total Index**			
At the initiation of treatment	0.85 (0.17)	0.90 (0.81 - 0.95)	<0.001
At the end of IP	0.92 (0.10)	0.95 (0.90 - 1.00)	
At the end of CP	0.95 (0.07)	0.95 (0.95 - 1.00)	
After 3 months of completion of treatment	0.96 (0.08)	1.00 (0.95 - 1.00)	
**Patient Health Questionnaire (PHQ)-9**			
At the initiation of treatment	5.09 (2.97)	5.00 (3.00 - 7.00)	<0.001
At the end of IP	2.41 (1.5)	2.00 (1.00 - 3.00)	
At the end of CP	1.25 (1.44)	1.00 (0.00 - 2.00)	
After 3 months of completion of treatment	0.88 (1.27)	0.00 (0.00 - 1.00)	
**Generalized Anxiety Disorder (GAD)-7**			
At the initiation of treatment	2.97 (1.72)	3.00 (2.00 - 4.00)	<0.001
At the end of IP	1.50 (1.25)	1.00 (1.00 - 2.00)	
At the end of CP	0.96 (1.06)	1.00 (0.00 - 1.00)	
After 3 months of completion of treatment	0.92 (1.06)	1.00 (0.00 - 1.00)	
**Short Form (SF)-20**			
At the initiation of treatment	66.01 (8.41)	66.48 (61.54 - 72.53)	<0.001
At the end of IP	76.29 (7.61)	76.92 (71.43 - 81.32)	
At the end of CP	82.11 (7.16)	82.42 (78.02 - 87.91)	
After 3 months of completion of treatment	83.41 (8.40)	85.71 (78.02 - 90.11)	
**St George’s Respiratory Questionnaire (SGRQ)**			
At the initiation of treatment	31.27 (17.50)	31.03 (16.94 - 43.04)	<0.001
At the end of IP	16.52 (10.60)	15.70 (9.15 - 22.02)	
At the end of CP	12.78 (9.01)	11.27 (5.65 - 18.52)	
After 3 months of completion of treatment	13.47 (9.15)	11.69 (7.52 - 16.67)	

#### General health.

The median scores of EQ-5D VAS (50% to 90%; p < 0.001), EQ-5D-5L index (0.9 to 1; p < 0.001), SF-20 (66.48% to 85.71%; p < 0.001) significantly increased from treatment initiation to after three months of completion of treatment period. The median SGRQ scores of the patients were reduced (31.03 to11.69; p < 0.001) after completion of treatment (Table 2).

#### Depression and anxiety.

The median PHQ-9 depression scores (5–0; p < 0.001) and GAD-7 anxiety scores (3–1; p < 0.001) of the patients significantly decreased after completion of treatment (Table 2).

### 3.4 Factors associated with General HRQoL using GEE

The HRQoL of the patients, as measured by the EQ-5D-5L scores, showed an improvement in general health over time (at the end of IP: 0.047, p < 0.05; at the end of CP: 0.067, p < 0.005; after three months of completion of treatment: 0.076, p < 0.001) compared to baseline. The EQ-5D-5L scores were lower in females (−0.038, p < 0.005) and higher in patients who did not skip the main meal in a day (0.077, p < 0.001). The general health SF-20 scores over time were lower in patients aged >45 years (−1.675, p < 0.05), females (−3.809, p < 0.001), and unemployed (−2.277, p < 0.005). In contrast, patients who were not skipping main meal had significantly higher scores (7.258, p < 0.001) indicating better general health. The general health status measured using SF-20 scores were improved during (at the end of IP: 6.535, p < 0.001; at the end of CP: 11.874, p < 0.001) and after three months of completion of treatment (12.941, p < 0.001) (Table 3). There is a marginal level (p = 0.07) of significance associated between EQ-5D-5L and literacy.

**Table 3:** GEE model for factor influencing HRQoL of TB patients using different tools over different time points.

Parameter	Patient Health Questionnaire (PHQ)-9		Generalized Anxiety Disorder (GAD-7)		Short Form (SF-20)		European Quality of Life-5 Dimensions-5 Level (EQ-5D-5L)		St George’s Respiratory Questionnaire (SGRQ)	
**B (SE)**	**p-value**	**B (SE)**	**p-value**	**B (SE)**	**p-value**	**B (SE)**	**p-value**	**B (SE)**	**p-value**
**Intercept**	5.766 (0.365)	<0.001	3.464 (0.224)	<0.001	63.743 (1.206)	<0.001	0.803 (0.024)	<0.001	32.328 (1.945)	<0.001
**District**										
Urban	1		1		1		1		1	
Rural	0.392 (0.162)	<0.05	0.121 (0.122)	0.318	−0.199 (0.633)	0.753	−0.012 (0.011)	0.298	2.219 (1.090)	<0.05
**Age in years**										
≤ 45 years	1		1		1		1		1	
>45 years	0.244 (0.183)	0.181	−0.074 (0.130)	0.569	−1.675 (0.716)	<0.05	−0.016 (0.012)	0.179	3.043 (1.128)	<0.01
**Gender**										
Male	1		1		1		1		1	
Female	0.609 (0.244)	<0.05	0.531 (0.186)	<0.005	−3.809 (1.053)	<0.001	−0.038 (0.013)	<0.005	4.256 (1.944)	<0.05
**Education**										
Illiterate	1		1		1		1		1	
Literate	−0.399 (0.207)	0.054	−0.103 (0.136)	0.451	1.337 (0.820)	0.103	0.027 (0.015)	0.070	−1.319 (1.189)	0.267
**Marital Status**										
Ever Married	1		1		1		1		1	
Never married	−0.065 (0.264)	0.807	−0.243 (0.185)	0.189	1.287 (0.942)	0.172	0.003 (0.015)	0.841	0.135 (2.005)	0.946
**Occupation**										
Employed	1		1		1		1		1	
Unemployed	0.112 (0.199)	0.574	0.146 (0.156)	0.349	−2.277 (0.738)	<0.005	−0.021 (0.012)	0.083	1.715 (1.389)	0.217
**Smoking habit**										
Smoker	1		1		1		1		1	
Non-smoker	−0.188 (0.212)	0.377	−0.151 (0.157)	0.336	1.266 (0.910)	0.164	0.010 (0.015)	0.519	−2.380 (1.458)	0.103
**Alcohol consumer**										
Yes	1		1		1		1		1	
No	−0.145 (0.225)	0.520	−0.104 (0.178)	0.559	0.779 (1.060)	0.462	0.020 (0.013)	0.121	0.758 (1.766)	0.668
**Skipping main meal in a day**										
Yes	1		1		1		1		1	
No	−1.669 (0.253)	<0.001	−1.011 (0.161)	<0.001	7.258 (0.937)	<0.001	0.077 (0.019)	<0.001	−8.413 (1.466)	<0.001
**Body Mass Index (kg/m^2^)**										
< 23	1		1		1		1		1	
≥23	−0.323 (0.197)	0.102	−0.190 (0.151)	0.209	2.000 (0.849)	<0.05	0.007 (0.013)	0.576	−1.113 (1.318)	0.398
**Follow-up Period**										
Initiation of Treatment	1		1		1		1		1	
At the end of Intensive Phase of treatment	−1.988 (0.280)	<0.001	−1.086 (0.174)	<0.001	6.535 (0.921)	<0.001	0.047 (0.019)	<0.05	−11.339 (1.439)	<0.001
At the end of Continuation Phase of treatment	−2.957 (0.301)	<0.001	−1.521 (0.190)	<0.001	11.874 (0.951)	<0.001	0.067 (0.019)	<0.005	−14.242 (1.628)	<0.001
After 3 months of completion of treatment	−3.298 (0.298)	<0.001	−1.548 (0.192)	<0.001	12.941 (1.118)	<0.001	0.076 (0.019)	<0.001	−13.104 (1.707)	<0.001

The disease specific SGRQ scores were decreased during (at the end of IP: −11.339, p < 0.001; at the end of CP: −14.242, p < 0.001) and after three months of completion of treatment (−13.104, p < 0.001) indicating a significant improvement in HRQoL compared to baseline. The scores were higher in patients aged >45 years (3.043, p < 0.01), females (4.256, p < 0.05) and those from rural district (2.219, p < 0.05) indicating that HRQoL was not improving significantly. However, patients who were not skipping their main meal had significantly lower SGRQ scores (−8.413, p < 0.001) indicating an improvement in HRQoL (Table 3).

### 3.5 Factors associated with depression and anxiety

The PHQ-9 depression scores and GAD-7 anxiety scores during follow up interview were significantly higher among females (0.609, p < 0.05; 0.531, p < 0.05). Additionally, the depression scores were also higher among patients from rural district (0.392, p < 0.05). In contrast, both depression and anxiety were lower among the patients who were not skipping their main meal (−1.669, p < 0.001; −1.011, p < 0.001). These scores were reduced over time from treatment initiation to post treatment. The estimates of depression scores during and after treatment were found to be statistically significant (at the end of IP: −1.988, p < 0.001; at the end of CP: −2.957, p < 0.001; after completion of treatment: −3.298, p < 0.001). Similarly the estimates of anxiety scores during and after treatment were also found to be statistically significant (at the end of IP: −1.086, p < 0.001; at the end of CP: −1.521, p < 0.001; after three months of completion of treatment: −1.548, p < 0.001). There was a marginally significant association between PHQ-9 scores and literacy (−0.399, p < 0.054).

## 4. Discussion

The salient finding of our study was the significant improvement in HRQoL of TB patients from treatment initiation to after three months of completion of treatment. Overall, the average HRQoL scores increased by 10–30% in all domains, indicating that the medical management of TB is effective not only in controlling the disease but also reducing its burden on patients. Similar findings demonstrating improvements in HRQoL among TB patients following treatment have been reported from various regions across the world [8,26–28]. Notably, post-treatment HRQoL scores among TB patients were found to be similar to those of the general population in India [29]. Furthermore, each year, about two million people with TB are treated successfully, resulting in a substantial number of life-years saved. In addition to these life-improving outcomes, our study findings provided additional benefits of the TB programme-particularly, the significant enhancement in patients’ HRQoL, shedding light on the impact of TB that extends beyond the clinical sphere.

In the current longitudinal study, the TB patients were assessed at multiple time points using the different domains of HRQoL measurement tools. Across all five tools, HRQoL among female TB patients was significantly lower compared to males. Similar findings have been reported in previous studies from various regions, indicating that women had lower physical health than men [29,30]. The reasons could be that females are highly sensitive to change in their health status and tend to have lower physical strength [31]. Lower HRQoL among women may stem from stigma, caregiving burdens, and limited access to care due to economic and social constraints, highlighting the need for gender-sensitive TB interventions. So far under NTEP, there is no gender specific interventions and there is a need to uptake gender sensitive interventions to improve the HRQoL. It was also reported that TB is associated with stigma, particularly affecting unmarried women who face difficulties in marriage prospects and married women who often fear about transmitting the disease to their children [32]. Gender plays an important role that stigma related to it could affect the psycho-social well beings of female TB patients. Gender could influence socioeconomic and cultural factors, which in turn limits the autonomy in health decision making, access and economic dependence which may affect the timely care, emotional well-being and overall HRQoL. In general, female TB patients may experience social isolation and discrimination, further impacting their HRQoL. A study from Pakistan also revealed that the TB patients of higher age, being female, low household income, and comorbidities were associated with poorer HRQoL among TB patients [33]. We reemphasize the need for a multifaceted approach that encompasses medical care, mental health support, social empowerment, gender-sensitive interventions, and community engagement to improve the HRQoL of female TB patients.

The other important finding from this study is that TB patients who skipped their main meal reported lower HRQoL scores compared to those who did not. It is well established that regular, nutritious and hygienic food is mandatory for improving HRQoL. A study conducted among poor people in India and South Africa revealed that the number of missed meals due to lack of resources inflated depression in adults [34]. Similarly, a prospective study from USA, adults aged >40 years showed that consuming only one meal per day was linked to an elevated risk of mortality [35]. In the context of TB, skipping meals or inadequate nutrition can result in malnutrition which may weaken the immune system and reduce patient’s ability to fight the infection effectively. To address this issue, currently NTEP is providing ₹1000 per month as a nutritional support to the patients till the completion of treatment. Additionally, the program also offers nutritional counselling not only to the patients to improve the treatment outcomes but also to their family members to aid in disease prevention.

Furthermore, the current study identified a negative association between age and HRQoL among TB patients. This correlation may be attributed to the natural decline in health that accompanies aging. These results are corroborated with studies conducted in various parts of the world that also showed the negative association between age and HRQoL [36,37]. The other reason is that as individuals age, their immune system may be weakened and making it more challenging to combat infections like TB. This can lead to a prolonged recovery periods and consequent decline HRQoL. In addition, older TB patients often have multiple co-morbidities which can complicate treatment and further reduce their overall well-being.

Another important finding from this study is that depression and anxiety scores significantly decreased from the baseline to post treatment. This aligns with earlier research recommendations emphasizing the need for psychological support to enhance TB treatment outcomes [3]. NTEP has already recognised this by integrating TB programme with mental health services so that it will improve the HRQoL particularly in reducing depression and anxiety. Subsequently NTEP has taken steps to strengthen the understanding and management of mental disorders among TB patients through increased advocacy, various patient support systems and promotion of patient to patient interactions [3]. These findings highlight the need for targeted psychosocial interventions and the involvement of community health workers to support patients with low baseline HRQoL, potentially improving their treatment adherence and overall well-being.

Over the course of treatment, the HRQoL scores were improved significantly. A similar trend was reported from a longitudinal study conducted in South Africa, where general health status improved significantly during the treatment period [7]. It is a well known fact that adherence to treatment, specifically taking the correct medication dosage at the right time, leading to improved HRQoL. Despite TB requiring prolonged treatment, patients in this study reported an improvement in their HRQoL over time. This positive outcome may be attributed to the efforts of NTEP, which has implemented various strategies to support treatment adherence. This includes the provision of monetary incentives, the use of digital technologies to monitor and promote regular medication intake [3]. Additionally, our findings revealed that literate patients had higher HRQoL compared to illiterate. Literacy of the patients is an important factor, as educated individuals are more likely to understand the importance of treatment adherence, which in turn promotes better adaptability, self-care and motivation-ultimately contributing to enhanced HRQoL.

In this study, we employed GEE, a robust statistical approach well suited for analysing HRQoL in longitudinal design. GEE offers flexibility in handling correlated observations, which is a key advantage when dealing with repeated measures data [25]. As a semi-parametric method, GEE was particularly effective in identifying factors influencing the HRQoL of TB patients. It also provides unbiased estimates, as drop-outs and patients who did not fully participate were treated as missing completely at random.

Our study findings should be interpreted in light of certain limitations. First, there is a possibility of recall bias due to the use of self-reported HRQoL measures. Second, the study specifically focused on newly diagnosed smear-positive drug-sensitive TB patients and the study excluded TB patients with drug resistant TB, recurrent TB, extra pulmonary TB and those co-infected with HIV. These excluded groups differ substantially in clinical characteristics, treatment duration and overall disease burden and their HRQoL trajectories are likely to differ significantly from the current study population. Therefore, the findings cannot be generalized to these patient groups. The HRQoL of these groups need to be studied separately.

## 5. Conclusion

Our findings suggest that the overall HRQoL including mental and emotional well-being of TB patients improved during and post treatment. This longitudinal study provides valuable insights for healthcare professionals and policymakers, highlighting the importance of integrating patient-centered care approach to further enhance the TB control. Additionally, routine monitoring of HRQoL may help prevent treatment lost-to-follow-up or relapse, especially among high-risk subgroups such as older adults, females, and individuals experiencing food insecurity.

## Supporting information

S1 Appendix Tools used in the study. (DOCX)

S2 File This is a minimal dataset for this analysis. (XLSX)
