# Supplementary material for: Health related quality of life of tuberculosis patients in South India: A longitudinal assessment study
Source: PLoS One. 2025 Jul 23;20(7):e0328484. doi: 10.1371/journal.pone.0328484 (PMC12286406; doi:10.1371/journal.pone.0328484)
Supplement: S1 Appendix — (DOCX) [file pone.0328484.s001.docx]

**S1 Appendix**

**Tools used in the study**

**European Quality of Life-5 Dimensions-5 Level (EQ-5D-5L):** The EQ-5D-5L is a widely-used standardized measure of general health status developed by the EuroQol Group. It consists of 5 item questionnaire about mobility, self-care, usual activities, pain/discomfort and anxiety/depression, each rated at 5 levels: no problems, slight problems, moderate problems, severe problems, extreme problems. The resulting health index ranges from 0 to 1, where 0 represents poor health and 1 represents best health. The EQ-VAS (Visual Analogue Scale) provides a quantitative measure of the patient’s perception of their current overall health, ranging from 0 to 100, where 0 represents poor health and 100 represents best health [1]. This tool is versatile and applicable across a wide range of diseases and conditions, allowing for comparisons across populations and interventions. Its main advantage lies in its simplicity and applicability in both clinical and non-clinical settings. The licenced version of EQ-5D-5L was obtained from the tool developer for use in this study.

**20-item Short Form Survey (SF-20): The** SF-20 measures six aspects of health status, comprising physical functioning (6 questions), role functioning (2 questions), social functioning (1 question), mental health (5 questions), current health perceptions (5 questions) and pain (1 question). The SF-20 scores range from 0 to 100, reflecting the health status. The lowest possible health score zero represents poor health whereas the highest possible health score 100 represents best health [2]. It was developed as part of the medical outcomes study and serves as an efficient tool for capturing overall health and well-being in a short format, making it suitable for use in large-scale studies or clinical evaluations.

**St George’s Respiratory Questionnaire (SGRQ):** The SGRQ is a disease-specific instrument developed to assess the quality of life in patients with respiratory diseases, such as tuberculosis (TB), chronic obstructive pulmonary disease (COPD), and asthma. The SGRQ has two parts designed to measure the health impairment in patients where the first part includes the symptoms score and second part comprises of the activity and impacts scores. The licenced version of SGRQ was acquired from the tool developer for this study. The part 1 consists of 8 questions that cover the patient’s recollection of their symptoms over a preceding period. Part 2 consists of 8 questions that address the patient’s current health state. Each response has a unique empirically derived 'weight'. The lowest possible weight is zero which indicates best health and the highest is 100 which indicates the poor health [3]. The SGRQ's specificity to respiratory health provides a more nuanced understanding of how lung disease affects HRQoL.

**Patient Health Questionnaire (PHQ-9):** The PHQ-9 is a widely-used self-report tool for screening, diagnosing and measuring the severity of depression. It is based on the criteria in the Diagnostic and Statistical Manual of Mental Disorders (DSM) and asks respondents to evaluate how frequently they have experienced the nine core symptoms of depression over the past two weeks. Depression is a significant comorbidity in many chronic diseases, including TB making its assessment essential for a comprehensive evaluation HRQoL. The scores of each question ranges from 0 to 3. Depression levels measured namely not at all, several days, more than half the days and nearly every day respectively. The total score of PHQ-9 ranges from 0 to 27, where 5, 10, 15 and 20 represents the cut points for mild, moderate, moderately severe and severe depression respectively [4].

**General Anxiety Disorder (GAD-7):** GAD-7 is an instrument for screening persistent worry and anxiety symptoms. It includes seven items that reflect the core symptoms of anxiety, such as nervousness, worry and difficulty in relaxing. Like depression, anxiety can severely affect patients' quality of life, particularly in chronic disease contexts, and its assessment is crucial for understanding the broader psychosocial impacts of disease. Anxiety levels are measured by assigning scores from 0 to 3 for each question, corresponding to the response categories: not at all, several days, more than half the days and nearly every day respectively. The total score for the seven items ranges from 0 to 21with scores of 5, 10, and 15 representing cut points for mild, moderate, and severe anxiety, respectively [5].

By combining these instruments, we can assess multiple facets of health, including general well-being, disease-specific issues, and mental health symptoms, providing a holistic view of the patient’s HRQoL. This approach ensures that both the physical and psychological impacts of TB and its treatment are thoroughly evaluated, which is critical for designing effective interventions and improving long-term patient outcomes.

**Reference**

[1] EuroQol. User guides. EuroQol. 2022. Available from:

<https://euroqol.org/publications/user-guides>

[2] Rcarver DJ, Chapman CA, Thomas VS, Stadnyk KJ, Rockwood K. Validity and reliability

of the Medical Outcomes Study Short Form-20 questionnaire as a measure of quality of life

in elderly people living at home. Age Ageing. 1999;28(2):169–74.

[3] Jones PW, Quirk FH, Baveystock CM. The St. George's Respiratory Questionnaire. Resp

Med 1991; 85 (suppl B):2531.

[4] Kroenke K, Spitzer RL, Williams JB. The PHQ-9: validity of a brief depression severity

measure. J Gen Intern Med. 2001;16(9):606–13.

[http://dx.doi.org/10.1046/j.1525- 1497.2001.016009606.x](http://dx.doi.org/10.1046/j.1525-%20%20%20%201497.2001.016009606.x)

[5] Spitzer RL, Kroenke K, Williams JBW, Löwe B. A brief measure for assessing generalized

anxiety disorder: the GAD-7: The GAD-7. Arch Intern Med. 2006;166(10):1092–7.

<http://dx.doi.org/10.1001/archinte.166.10.1092>
